# Association of conventional ultrasound, elastography and clinicopathological factors with axillary lymph node status in invasive ductal breast carcinoma with sizes > 10 mm

**DOI:** 10.18632/oncotarget.18969

**Published:** 2017-07-04

**Authors:** Hui Liu, Guang Xu, Ming-Hua Yao, Huan Pu, Yan Fang, Li-Hua Xiang, Rong Wu

**Affiliations:** ^1^ Department of Medical Ultrasound, Shanghai Tenth People's Hospital, Ultrasound Research and Education Institute, Tongji University School of Medicine, Shanghai 200072, China

**Keywords:** conventional ultrasound, elastography, clinicopathological factors, axillary lymph node status, invasive ductal breast carcinoma

## Abstract

**Background:**

To evaluate whether conventional ultrasound, elastography [conventional strain elastography of elasticity imaging, acoustic radiation force impulse induced strain elastography of virtual touch tissue imaging, and a novel two-dimensional shear wave elastography of virtual touch tissue imaging quantification] and clinicopathological factors are associated with axillary lymph node metastasis in invasive ductal breast carcinoma with sizes > 10 mm.

**Materials and Methods:**

We evaluated 150 breast lesions from 148 patients using the above methods and the clinicopathological factors. Univariate and multivariate logistic regression analysis were performed to determine the axillary lymph node metastasis risk factors. Diagnostic performance was evaluated using receiver operating characteristic curve analysis.

**Results:**

Sixty-three tumors (42%) were node-positive, 87 (58%) were node-negative. Aspect ratio, virtual touch tissue imaging grade, shear wave velocity, pathological invasive tumor size, and histological grade maintained independent significance in predicting nodal involvement. The mean tumor shear wave velocitys (4.60, 6.49, 7.16) increased in proportion to metastatic node number (0, 1–3, ≥ 4, respectively; *P* < 0.001). For all tumors in this study, the cut-off shear wave velocity was 6.16 m/s and was associated with 64.1% sensitivity, 78.0% specificity and an area under the ROC curve of 0.799 (95% confidence interval, 0.731–0.868).

**Conclusions:**

Aspect ratio, virtual touch tissue imaging grade, shear wave velocity, pathological invasive tumor size and histological grade are independently associated with axillary lymph node metastasis in invasive ductal breast carcinoma with sizes > 10 mm.

## INTRODUCTION

Breast cancer is the most frequent cancer in women worldwide [1]. The nodal status of patients with breast cancer at initial clinical diagnosis is generally considered one of the most important factors for prognosis [2, 3]. Prognosis and therapeutic decisions are guided by the assessment of metastatic axillary lymph nodes [4]. The sentinel lymph node (SLN) is the first lymph node to receive lymph drainage from the primary tumor and is also highly predictive of the status of the remaining axillary lymph nodes [5, 6]. If no metastatic axillary lymph nodes are detected, the standard procedure is sentinel lymph node biopsy (SLNB), but if the finding is positive, then the patient has to undergo a second surgical procedure for axillary lymph node dissection (ALND) [7]. ALND is associated with more complications, including lymphedema, arm paresthesia, chronic pain, and immobility, and ALND alone does not improve survival in breast cancer [8–10]. SLNB has been incorporated into standard guidelines as an appropriate initial alternative to ALND in patients with clinically node-negative breast cancer [11]. Although SLNB is accurate for predicting axillary lymph node status, up to 15% false negative results have been reported, where other associated nodes in the same region were positive [9]. The detection of a metastatic lymph node prior to surgery enables immediate ALND without performing SLNB first [12, 13]. Avoiding a non-useful SLNB saves time and money and reduces the risk for post-surgical complications [13]. Therefore, accurate prediction of axillary nodal status using a noninvasive imaging technique at this juncture would be of great value in such patients to avoid unnecessary axillary surgery, including SLNB.

Ultrasound (US) is frequently used for preoperative assessment of patients with breast cancer and may be well-positioned to detect possible metastatic axillary lymph nodes and reduce the number of false negatives from SLNB. The value of ultrasonography in diagnosing breast lesions has been confirmed: morphological features of the lesions are displayed clearly, and the diagnostic sensitivity and specificity are > 90% [14]. Axillary sonography is moderately sensitive and fairly specific for diagnosing axillary metastatic involvement, as visualizing the relatively deep axillary lymph nodes on US is difficult, and in metastatic lymph nodes without typical morphological changes, US cannot distinguish between benign and malignancy [15].

At present, a new imaging technique uses ARFI technology to yield information not only on the morphological characteristics, but also quantitative measurements of the mechanical stiffness properties (elastic) of tissue [16, 17]. Recently, Evans et al. showed that breast cancer stiffness measured by SWE was associated with lymph node metastasis on univariate analysis [18].

Invasive breast cancers are a histologically heterogeneous group of lesions. More recently, some authors have sought to gain information on the heterogeneous tumor biology of breast cancer from US images and elastography, and concluded that tumor elastography values are associated with traditional prognostic factors such as tumor size, histological grade, and axillary lymph node involvement. Conventional US or elastography findings could independently predict lymph node status when taking known predictors of nodal status such as invasive size and histological grade into account [19, 20]. However, the results have not been consistent across studies and the association between tumor elastography values and ALNM has not been fully determined, especially in early-stage disease. The aim of this study was to determine, in a large series of patients with invasive breast cancer treated initially by surgery, whether conventional US, elastography and clinicopathological factors are associated with ALNM.

## RESULTS

### Histopathological findings

Supplementary Table 1 shows the B-mode US, elastography imaging and histological characteristics of the study group. Histopathological examination confirmed that all 150 tumors were invasive ductal carcinoma. Sixty-three tumors (42%) were node-positive, 87(58%) were node-negative at surgical histopathology (Supplementary Table 1). Pathological invasive tumor size ranged 12–90 mm. The mean US size of the tumors was not significantly different from their mean pathological tumor size (25.32 ± 14.48 mm vs. 25.84 ± 14.47 mm; *P* > 0.05).

### Univariate logistic regression analysis

Among the conventional US, elastography imaging and clinicopathological variables, univariate logistic regression analysis demonstrated that predictors such as US size (OR: 1.798, *P* = 0.031), aspect ratio (OR: 2.462, *P* = 0.009), calcification (OR: 1.612, *P* = 0.019), Adler grade of blood flow (OR: 2.422, *P* = 0.010), elasticity score (OR: 2.206, *P* < 0.001), VTI grade (OR: 2.173, *P* < 0.001), SWV (OR: 1.984, *P* < 0.001), pathological invasive tumor size (OR: 1.849, *P* = 0.013) and histological grade (OR: 3.957, *P* < 0.001) were significantly different between node-positive and node-negative tumors. None of the reference parameters such as location, distance from skin, number, shape, margin, background echotexture, posterior features, BI-RADS category, age, ER, PR, HER-2 or Ki-67 were statistically significant in predicting ALNM in invasive ductal breast carcinoma with sizes > 10 mm (all *P* > 0.05) (Supplementary Table 1).

The incidence of ALNM was 0% (0 of 0), 23.5% (4 of 17), 13.6% (3 of 22), 36.0% (18 of 50), and 62.3% (38 of 61) in tumors with an elasticity score of 1, 2, 3, 4, and 5, respectively (Supplementary Table 1). ALNM was significantly more frequent in tumors with high elasticity scores than in tumors with low elasticity scores [50.5% (56 of 111) vs. 17.9% (7 of 39); *P* < 0.001] (Supplementary Table 1). Likewise, ALNM was significantly more frequent in tumors with VTI grade classified as pattern 4b [83.3% (20 of 24)] (Supplementary Table 1). The NPV of low elasticity scores and VTI grade pattern 1 for ALNM was 82.1% (32 of 39) and 82.6% (19 of 23), respectively, in 150 tumors.

### Multivariate logistic regression analysis

Multivariate logistic regression analysis entering all independent variables that were significant in univariate logistic regression analysis was performed and determined that predictors such as aspect ratio (OR: 5.259, *P* = 0.003), VTI grade (OR: 2.294, *P* < 0.001), SWV (OR: 1.873, *P* < 0.001), pathological invasive tumor size (OR: 3.121, *P* = 0.004) and histological grade (OR: 3.973, *P* = 0.010) were independently associated with ALNM while US size, calcification, Adler grade of blood flow and elasticity score were not (Table 1).

**Table 1:** Multivariate logistic regression analysis for predicting ALNM

Dependent variable	Independent variable	B	SE	Wald	df	*P*-value	Odds ratio	95% CI
Nodal involvement (yes/no)	**US size**					0.450		
	**Aspect ratio**	1.660	0.559	8.810	1	0.003	5.259	1.757–15.735
	**Calcification**					0.067		
	**Adler grade of blood flow**					0.318		
	**Elasticity score**					0.380		
	**VTI grade**	0.830	0.205	16.343	1	< 0.001	2.294	1.534–3.430
	**SWV values**	0.627	0.146	18.573	1	< 0.001	1.873	1.408–2.491
	**Pathological invasive tumor size**	1.138	0.392	8.414	1	0.004	3.121	1.446–6.735
	**Histological grade**	1.380	0.533	6.691	1	0.010	3.973	1.397–11.301

**Figure 1:**
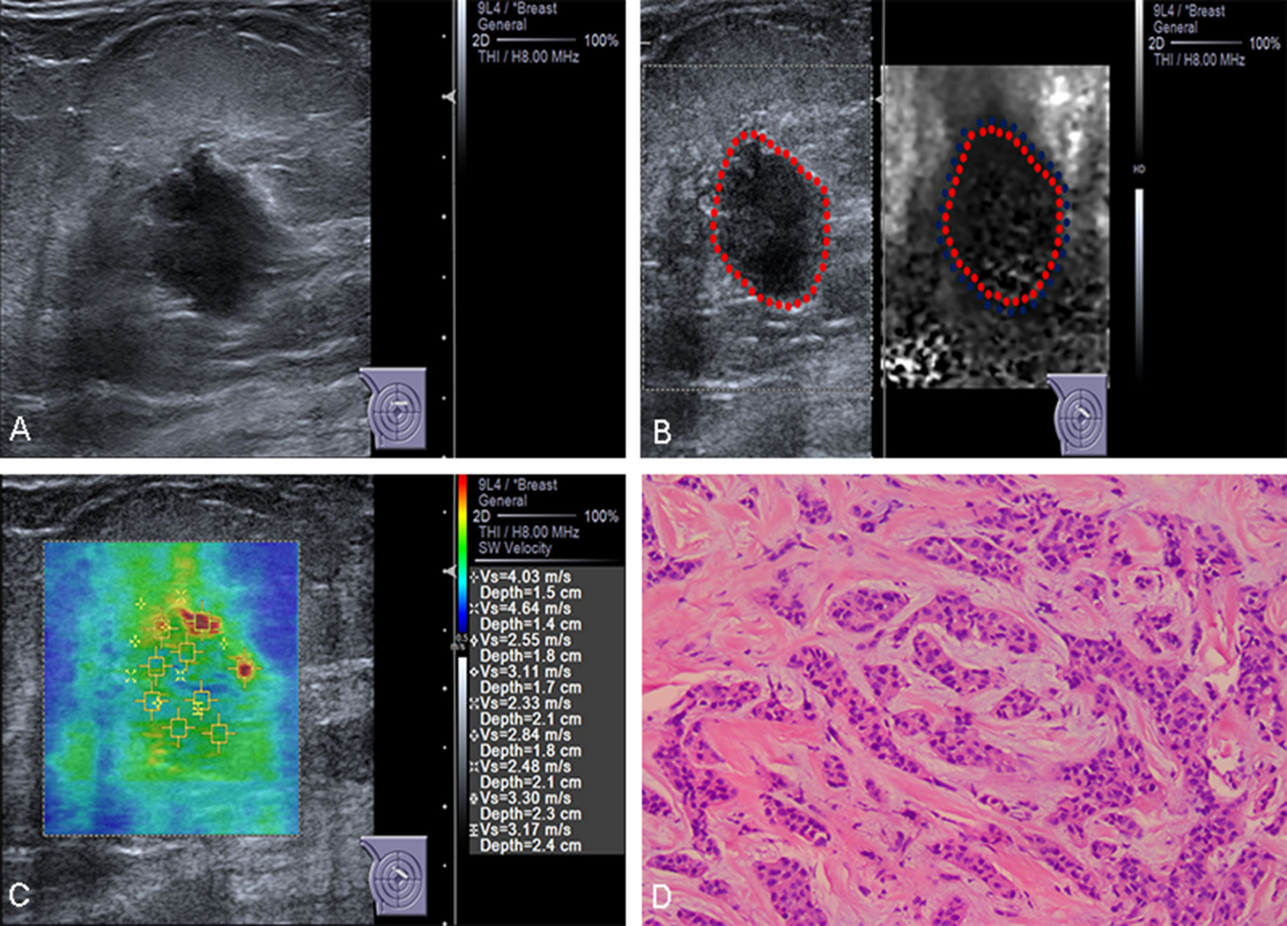
Conventional US and ARFI elastography of a 83-year-old female patient (**A**) Conventional US image shows a 15-mm invasive ductal carcinoma with aspect ratio > 1. (**B**) VTI image shows that the VTI grade is classified as pattern 4a. The lesion contains both bright and dark areas, the dark area corresponds in size to the lesion on the B-mode US image (lineblue/linered). (**C**) ROIs are marked within the lesions. SWVs are shown on the novel 2D-SWE of VTIQ image. The mean SWV is 3.16 m/s. (**D**) Pathologic confirmation was of an invasive ductal carcinoma, histological grade 2, with node-negative findings.

**Figure 2:**
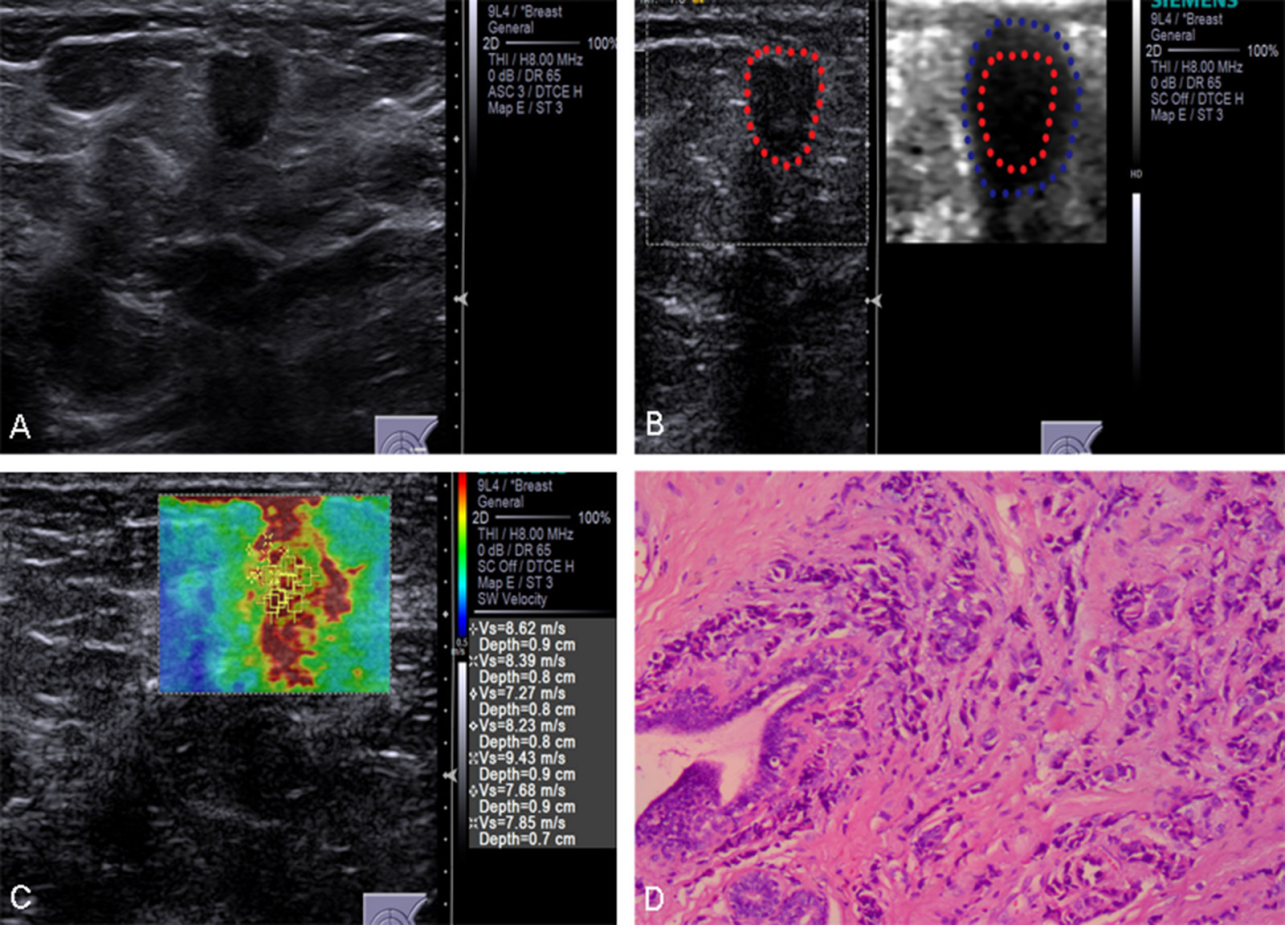
Conventional US and ARFI elastography of a 63-year-old female patient (**A**) Conventional US image shows a 13-mm invasive ductal carcinoma with aspect ratio > 1. (**B**) VTI image shows that the VTI grade is classified as pattern 4b. The dark area is larger than the lesion on the B-mode US image (lineblue/linered). (**C**) ROIs are marked within the lesions. SWVs are shown on the novel 2D-SWE of VTIQ image. The mean SWV is 8.15 m/s. (**D**) Pathologic confirmation was of an invasive ductal carcinoma, histological grade 2, with node-positive findings.

Univariate and multivariate logistic regression were used to establish the significance of the associations of B-mode US, elastography imaging and histological findings with lymph node status. Aspect ratio, VTI grade, SWV, pathological invasive tumor size and histological grade maintained independent significance in predicting nodal involvement (Figures 1 and 2 ).

Table 2 shows the tumor stiffness values according to nodal status. The mean tumor SWVs increased in proportion to the number of metastatic nodes (0, 1–3, and ≥ 4) in 150 tumors (4.60, 6.49, and 7.16, respectively; *P* < 0.001), particularly for pathological invasive tumor size ≤ 50 mm (Table 2). ROC curves were constructed to determine the optimal cut-off value for predicting ALNM. For all tumors (axillary status–positive and -negative), the cut-off SWV was 6.16 m/s, and it was associated with 64.1% sensitivity, 78.0% specificity, and an area under the ROC curve (AUC) of 0.799 (95% CI, 0.731–0.868) (Table 3).

**Table 2:** VTI grade and SWV values of tumors according to pathological invasive tumor size and number of metastatic nodes

Pathological invasive tumor sizes (mm)	Number of metastatic nodes	VTI grade		Range of SWV values (m/s)	Median of SWV values (m/s)	Mean of SWV values (m/s)	P^*^
		1,2,3,4a	4b				
Total	0 (*n* = 87)	83 (65.9)	4 (16.7)	0.75–8.78	4.63	4.60	< 0.001
	1–3 (*n* = 36)	21 (16.7)	15 (62.5)	4.00–9.47	6.82	6.49	
	≥ 4 (*n* = 27)	22 (17.4)	5 (20.8)	4.63–9.68	7.00	7.16	
≤ 20	0 (*n* = 51)	48 (77.4)	3 (25.0)	0.75–8.50	3.97	4.38	< 0.001
	1–3 (*n* = 16)	8 (12.9)	8 (66.7)	4.00–8.40	6.83	6.35	
	≥ 4 (*n* = 7)	6 (9.7)	1 (8.3)	5.57–8.64	6.30	6.75	
20–50	0 (*n* = 28)	27 (54.0)	1 (10.0)	2.00–8.00	4.96	4.75	< 0.001
	1–3 (*n* = 14)	11 (22.0)	5 (50.0)	4.00–9.48	7.32	6.73	
	≥ 4 (*n* = 16)	12 (24.0)	4 (40.0)	4.63–9.68	7.29	7.58	
> 50	0 (*n* = 8)	8 (50.0)	0 (0)	4.00–8.79	5.16	5.47	0.599
	1–3 (*n* = 6)	4 (25.0)	2(100)	5.00–8.70	5.46	6.29	
	≥ 4 (*n* = 4)	4 (25.0)	0(0)	5.17–8.40	5.63	6.21	

^*^SWV values of different size groups were analyzed using one-way ANOVA.

Numbers in parentheses are percentages.

**Table 3:** Diagnostic performance of VTIQ parameters in differentiating axillary lymph node involvement

	Cut-off value (m/s)	AUC (%)	Sensitivity (%)	Specificity (%)	Accuracy (%)	PPV (%)	NPV (%)
SWV-mean	6.16	0.799 (0.731–0.868)	64.1%	78.0%	72.0%	68.3%	74.4%

AUC, area under the ROC curve; PPV, positive predictive value; NPV, negative predictive value.

## DISCUSSION

Despite advances in diagnosis and treatment, breast cancer remains the second leading cause of death of women in the developed world [27]. Numerous clinicopathological factors and novel molecular markers have been investigated to improve the prediction of clinical outcome for patients with breast cancer. In the clinical pathology setting, when there are 0, 1–3, 4–9, or ≥ 10 involved lymph nodes, the cancer may be classified as N0, N1, N2, or N3, respectively [24]. Lymph node status is one of the most important factors for determining a patient's prognosis, and knowledge of lymph node status influences both surgical management and the treatment plan [28]. However, conventional US may not identify the axillary lymph nodes preoperatively if they are small. Previous SWE studies have found that increased stiffness is associated with nodal metastases on univariate analysis [18, 29]. The present study included US features and elastography features as potential predictors of ALNM. The results indicate that ALNM is dependent on features such as aspect ratio, VTI grade, SWV, pathological invasive tumor size and histological grade. The same as previous studies, histological grade was an independent predictor of nodal involvement on multivariate analysis. High histological grade was also associated with increased stiffness [29]. This may be particularly useful because conventional US findings in high-grade cancers can be less characteristic than those in low-grade cancers [30]. Aitken and Osman also proved that ALNM risk is lower when the breast tumor is small (diameter < 2 cm), and tumor size is an independent predictor of nodal positivity [31]. Due to the larger size, a larger area of adjacent tissues invaded by the cancer indicates a higher risk for lymph node metastasis.

As a noninvasive technique, elastography, including conventional SE of elasticity imaging, ARFI-induced SE of VTI and the novel 2D-SWE of VTIQ, is a complementary tool for conventional US to evaluate tissue hardness. The present study has indicated that pathological findings corresponding to the dark region of increased size on VTI are node-positive in most malignant lesions. We also divided pattern 4 lesions into patterns 4a and 4b, as did Garra et al. [32] and Tozaki et al. [22]. The subdivision is important because we found that ALNM was more common in pattern 4b lesions. Nevertheless, the width difference of pattern 4 lesions between ARFI and B-mode images could be used for predicting ALNM. Given the peritumoral invasion in malignant lesions, ALNM in invasive breast cancers would probably display pattern 4b on ARFI elastography. For breast cancer with poorly defined margins (i.e., ill-defined, spiculated, hyperechoic halo, microlobulated, angulated), VTI examination is necessary for assessing the extent of cancer invasion and ALNM. This can reduce the rate of ALNM misdiagnosis.

The novel 2D-SWE of VTIQ can provide new insights into the evaluation of tissue characteristics in a noninvasive fashion. Positive correlation of tissue stiffness with VTIQ velocity has also been demonstrated. VTIQ could also classify the biological features of the tissues according to SWV quantity. The wave propagation speed generally represents an intrinsic and reproducible property of a given tissue. Unlike conventional elastography that is based on mechanically induced deformation (strain), ARFI is operator-independent, reproducible, and quantitative. The VTIQ velocity generated by ARFI is therefore an objective and reproducible datum of the intrinsic tissue characteristics. The larger of the previous studies used only one shear wave measurement from each tumor; in the study, we removed the maximum and minimum values from the seven measurements for VTIQ, then calculated the mean values, further increasing the statistical power of our study.

Our results demonstrate that ALNM has significantly higher VTIQ velocities than in non-metastasis. In addition, there was a statistically significant difference in VTIQ velocities among the number of lymph node metastasis. The mean VTIQ velocities of tumors increased in proportion to the number of metastatic nodes in the 150 tumors, particularly for pathological invasive tumors ≤ 50 mm. For the tumors > 50 mm, liquefaction necrosis could easily occur in the central of the lesion. It maybe have an effect on SWV. There was a large variation in the mean VTIQ velocities within each tumor size group, suggesting that intrinsic tumor biological factors cause variations in tumoral and peritumoral stiffness. This is therefore the first study to examine the correlation between VTIQ velocity and axillary lymph node status. Therefore, VTIQ is considered to have the potential to diagnose invasive ductal breast cancer metastasis of the axillary lymph nodes. We also examined the optimal cut-off value for VTIQ to distinguish non-metastasis from metastasis using the ROC method. The results demonstrate that the best cut-off value for the velocity for distinguishing non-metastatic from metastatic lymph nodes is 6.16 m/s. There is a high possibility of ALNM if the VTIQ velocity > 6.16 m/s. The reliability of the multivariate analysis was confirmed by the AUC of 0.799. VTIQ is therefore a potential procedure for reducing unnecessary axillary lymph node biopsy in patients with invasive ductal breast cancer.

Conventional US and SWE may therefore be complementary in identifying ALNM in invasive ductal breast cancers. In the present study, hard tumors, as indicated by VTI grade and SWV, were associated with ALNM. These findings are in agreement with a previous study [33] that showed that lesion stiffness is an independent predictor of ALNM in women with invasive breast cancer.

The stiff tissue associated with invasive breast cancer is usually seen at the periphery of the tumor, extending into the peritumoral stroma. Often, the tumor itself is less stiff than the surrounding stroma. Although not proven, it is likely that the stiffness represents abnormal tumor-associated collagen, which exhibits increased crosslinking and abnormal alignment. These collagen abnormalities have recently been shown to have independent prognostic significance [34, 35]. It is possible that the peritumoral stromal stiffness represents an imaging surrogate for some of these stromal processes. As these stromal prognostic indicators are not measured by the conventional predictors of nodal involvement, such as invasive tumor size and histological grade, it is not surprising that stromal stiffness has independent predictive significance.

Preoperative diagnosis of nodal metastases is valuable, as it refines preoperative prognostication when added to aspect ratio, VTI grade, SWV estimation on imaging, pathological invasive tumor size, and histological grading on ALND or SLNB. Previous studies have drawn similar conclusions [36, 37]. This can facilitate the use of neoadjuvant chemotherapy (NACT) to downstage tumors prior to surgery or to identify more accurately at diagnosis the most appropriate axillary surgery for patients following NACT. Our findings suggest that elastography (VTI and VTIQ) has the potential to refine preoperative prognostication and therefore aid in improving decision-making with regard to NACT and management of the axilla.

This study has several limitations. The main weakness is that it is from a single center. We do not know whether such results are reproducible at other clinical centers. The number of patients in the present study is relatively small but the results still suggest the potential value of VTIQ examination combined with conventional US in the clinical management of breast cancer axillary lymph node status. Second, as a single-institution retrospective analysis, there was inevitable selection bias. Third, inter- and intraobserver variability in data acquisition and interpretation was not assessed. Finally, we did not establish a rating system using a risk model that included conventional US and ARFI elastography for predicting ALNM in invasive breast cancer. Further investigations employing more patients with longer clinical follow-up may be required to refine the benefits of elastography for ALNM of breast cancer in many situations.

In our analysis, aspect ratio, VTI grade, SWV, pathological invasive tumor size and histological grade are independently associated with ALNM in invasive breast cancer. VTI and VTIQ have potential as predictive markers of ALNM and could help in the selection of therapeutic strategies regarding axillary node management. Consequently, this can contribute additional noninvasive prognostic information as compared to conventional preoperative tumor assessment and staging, and avoid unnecessary axillary surgery including SLNB.

## MATERIALS AND METHODS

### Patients

The sample comprised images from 148 patients with 150 breast lesions that were confirmed between June 2014 and July 2015 as invasive ductal cancers at the Tenth People's Hospital of Tongji University. The inclusion criteria were: (1) The patient had undergone primary surgical treatment and ALND; (2) The lesion was confirmed as invasive ductal carcinoma by histopathology; (3) There were screen-detected abnormalities; (4) The B-mode US, elastography imaging and clinicopathological variable data were complete. Patients were excluded if: (1) The breast had undergone previous surgery; (2) Pathology confirmed other tumors in addition to invasive ductal carcinoma; (3) The patient had undergone the treatment such as chemotherapy and radiotherapy; (4) The patient had distant metastasis. The lesion diameters ranged 12–90 mm. The patient ages ranged 26–92 years; the mean age was 56.94 ± 12.27 years. Sixty-three patients (42%) had lymph node metastases. The lesions were visible on the US scans. We obtained B-mode US examinations routinely and elasticity imaging at the diagnostic breast clinic. In addition, a single experienced pathologist made all pathological diagnoses.

In accordance with the ethical and scientific review board of the Tenth People's Hospital of Tongji University, ethical approval for the study was not required.

### B-mode US and elastography examinations

Conventional US and elastography examinations were performed using a Siemens Acuson S3000 Ultrasound System. A linear array transducer 10L12 with 4–9-MHz multi-frequency was used for conventional US examination; a 4–9-MHz multi-frequency 9L4 transducer (Acoustic Radiation Force Impulse, Virtual Touch IQ; Siemens) was used for VTIQ examination. The SWE mode was available on a Siemens S3000 Ultrasound System equipped with VTIQ software. Both conventional US and elastography examination were performed by a single consultant specialist with at least 15 years’ experience in breast imaging and at least 3 months’ experience performing elastography examinations of breast lesions. The patient lay supine with the breast and axillary glands fully exposed. The conventional US device scanned the breast radially from the center starting with the nipples. Information on lesion size, location (upper outer quadrant, upper inner quadrant, lower outer quadrant, lower inner quadrant, subareolar), distance from skin, number (single or multiple), shape (oval or round, or irregular), margin (circumscribed, indistinct, angular, microlobulated, spiculated), background echotexture (homogeneous or heterogeneous), posterior features (none, enhancement shadowing), aspect ratio (< 1 or ≥ 1), calcification (none, coarse calcification, microcalcification), and color Doppler were recorded. In addition, the color Doppler flow patterns were defined as follows: type I, absence of visible flow; type II, peripheral flow without internal flow; type III, rare internal flow with or without peripheral flow; and type IV, rich internal flow with or without peripheral flow. Radial and anti-radial scanning was performed for accurate characterization, and the final assessment category according to the Breast Imaging Reporting and Data System (BI-RADS) lexicon was determined as follows [21]: category 1, negative findings; category 2, benign findings; category 3, probably benign findings; category 4, findings suspicious for malignancy; category 5, findings highly suggestive of malignancy. Category 3 lesions had < 2% probability of malignancy. Category 4 lesions had 3–94% probability of malignancy and were subcategorized into 4a, 4b, or 4c. Category 4a lesions had 3–10% probability of malignancy. Category 4b and 4c lesions had 11–50% and 51–94% probability of malignancy, respectively. Category 5 lesions had > 95% probability of malignancy. Category 4 and 5 lesions were recommended for biopsy. Although category 3 lesions were recommended for follow-up imaging, biopsies were performed upon clinician or patient request.

The planes of maximum diameters were selected for elastography assessment. For elasticity imaging assessment, the target lesion was vertically compressed by the transducer with light pressure. The target lesion was then displayed with color mapping according to the level of strain, ranging from red (softest component) to green (intermediate stiffness) to blue (hardest component). Referencing Itoh et al. [16] the breast lesions were differentiated into five categories according to color pattern. A score of 1 indicated equivalent strain throughout the lesion (no blue voxels were present in the lesion); a score of 2 indicated strain in most of the lesion with some areas without strain (a mosaic pattern of blue and other colors); a score of 3 indicated no strain in the central part of the lesion (only the central part of the lesion was blue); a score of 4 indicated no strain throughout the lesion (the entire lesion was blue); a score of 5 indicated no strain throughout the lesion or the surrounding area (the entire lesion and its surrounding area were blue).

After activating VTI or VTIQ, the transducer was gently placed onto the skin with no pressure and kept stationary, and the patient was asked to hold her breath during the acquisition of shear wave velocities (SWVs) and VTI images. In VTI mode, the VTI images and grayscale US images were displayed simultaneously in split-screen mode. The VTI image grayscale was classified into two values (bright and dark) through comparison between the mass and the surrounding breast tissue (the pectoral muscle and rib were excluded). To classify VTI mode images, the correspondence of lesions on ARFI elastographic images to those on B-mode images was evaluated. Lesions on B-mode images that failed to be visually confirmed on ARFI images were classified as pattern 1. Among the lesions that could be visually confirmed, those that were bright were classified into pattern 2; those that were dark into pattern 4. Lesions that contained both bright and dark areas were classified into pattern 3. In addition, pattern 4 was subdivided into 4a (dark area corresponding in size to the lesion on the B-mode image) and 4b (dark area larger than the lesion on the B-mod image) [22].

In the VTIQ mode, a novel two-dimensional quantitative SWE (2D-SWE) and color-coded qualitative and quantitative maps developed with SWVs were used. With the adjacent color spectrum as reference, red and green areas corresponded to high and low relative SWVs ranging between 0.5 and 10 m/s, respectively [23]. The regions of interest (ROIs) were marked within the lesions, avoiding calcified areas, cystic areas, and necrotic tissue. The elasticity values were obtained by moving a delineated ROI over the color map. As the ROI moved, the readings changed in real time, so the ROI could be positioned over the part of the image showing the stiffest tissue. ROIs were defined by the sonographer, and SWVs for the targeted area were measured. Qualitative maps of shear waves that displayed green for SWVs were considered reliable. For each effective measured area under the VTIQ velocity color overlay, we obtained seven repeat measurements for each lesion to test the retest reliability of VTIQ, then removed the maximum and minimum values, calculated the mean values for analysis.

### Histopathological analysis

All patients underwent surgical resection of breast cancer with ALND. Macro- (> 2 mm) and micrometastases (0.2–2.0 mm) were considered “positive” results; isolated tumor cells (≤ 0.2 mm) were considered a “negative” result. Tumor-node-metastasis staging was determined according to the American Joint Committee on Cancer (AJCC), 7th edition [24]. The tumor histological grades were assessed using the Nottingham combined histologic grade system. The expression statuses of estrogen receptor (ER), progesterone receptor (PR), human epidermal growth factor receptor 2 (HER2), and Ki-67 were determined based on the surgical specimens using the avidin-biotin complex immunohistochemical technique. The Allred score, based on staining proportion and staining intensity of positive cells, was used to evaluate the ER and PR status [25]. Tumors with an Allred score of at least 3 were considered positive. HER2 staining intensity was graded as 0, 1+, 2+, and or 3+ [26]. Tumors scoring 3+ were considered HER2-positive; tumors scoring 0 or 1+ were considered HER2-negative. Tumors that scored 2+ were evaluated further by fluorescence *in situ* hybridization (FISH). If the ratio of the *HER2* gene signal to the chromosome 17 probe signal was > 2.2, the tumor was classified as HER2-positive. For Ki-67, nuclear staining ≥ 14% was considered high-level expression.

### Statistical analysis

Statistical analysis was performed with SPSS software (version 17.0; SPSS Inc., Chicago, IL). Patients were divided into two groups according to the presence or absence of ALNM. Continuous quantitative data are expressed as the mean ± standard deviation (SD) if in normal distribution and as the range if otherwise. The mean US size of the 150 tumors was compared with their mean pathological tumor size using a paired Student's *t*-test. Categorical and continuous variables in two groups were analyzed using univariate logistic regression. Univariate analysis was used to analyze the correlation between the predictive factors and ALNM. Multivariate logistic regression analysis was performed to determine the risk factors for ALNM. Odds ratios (ORs) with 95% confidence intervals (CIs) were calculated. Univariate and multivariate logistic regression were used to establish the significance of the associations of B-mode US, elastography imaging and clinicopathological findings with lymph node status. The association among VTI grade, tumor SWV and axillary nodal status was evaluated according to pathological invasive tumor size and number of metastatic nodes. Pathological invasive tumor size was divided into three groups (≤ 20 mm, 20–50 mm, > 50 mm); the numbers of metastatic nodes were divided into 0, 1–3, and ≥ 4, respectively, for analytic purposes. The SWVs of groups of different sizes were analyzed using one-way analysis of variance (ANOVA). Receiver operating characteristic (ROC) analyses were performed to assess the diagnostic performance of 2D-SWE of VTIQ in differentiating ALNM from non-metastasis in invasive breast cancer. ROC analyses were also used to determine the best diagnostic cut-off value for VTIQ, and the corresponding sensitivity, specificity, accuracy, positive predictive value (PPV) and negative predictive value (NPV) were calculated for this diagnostic cut-off. *P*-values < 0.05 were considered to indicate a significant difference.

## SUPPLEMENTARY MATERIALS TABLE





## Abbreviations

SE: strain elastography
EI: elasticity imaging
ARFI: acoustic radiation force impulse
VTI: virtual touch tissue imaging
VTIQ: virtual touch tissue imaging quantification
2D-SWE: two-dimensional shear wave elastography
SWV: shear wave velocity
ALNM: axillary lymph node metastasis
SLN: sentinel lymph node
SLNB: sentinel lymph node biopsy
ALND: axillary lymph node dissection
BI-RADS: Breast Imaging Reporting and Data System
NACT: neoadjuvant chemotherapy
ROC: receiver operating characteristic
AUC: area under the ROC curve
ORs: Odds ratios
CIs: confidence intervals
PPV: positive predictive value
NPV: negative predictive value.
