# Supplementary material for: Association of conventional ultrasound, elastography and clinicopathological factors with axillary lymph node status in invasive ductal breast carcinoma with sizes > 10 mm
Source: Oncotarget. 2017 Jul 4;9(2):2819–28. doi: 10.18632/oncotarget.18969 (PMC5788682; doi:10.18632/oncotarget.18969)
Supplement: Supplementary file 1 [file oncotarget-09-2819-s001.pdf]

Association of conventional ultrasound, elastography and clinicopathological factors with axillary lymph node status in invasive ductal breast carcinoma with sizes > 10 mm

SUPPLEMENTARY MATERIALS

Supplementary Table 1: B-mode US, elastography imaging and clinicopathological variables of invasive ductal carcinoma and axillary nodal status (univariate logistic regression analysis). See_Supplementary_Table 1
