# Supplementary material for: Association of conventional ultrasound, elastography and clinicopathological factors with axillary lymph node status in invasive ductal breast carcinoma with sizes > 10 mm
Source: Oncotarget. 2017 Jul 4;9(2):2819–28. doi: 10.18632/oncotarget.18969 (PMC5788682; doi:10.18632/oncotarget.18969)
Supplement: Supplementary file 2 [file oncotarget-09-2819-s002.docx]

**Table 1.** B-mode US, elastography imaging and clinicopathological variables of invasive ductal carcinoma and axillary nodal status (univariate logistic regression analysis)

| Reference parameter | | Total  (n = 150) | Axillary status | | OR | P |
| --- | --- | --- | --- | --- | --- | --- |
|  |  |  | Positive (n= 63) | Negative (n=87) |  |  |
| **B-mode US** | |  |  |  |  |  |
| **US size**  (mean ± SD; mm) | | 25.32 ± 14.48 | 28.00 ± 16.73 | 23.39 ± 12.35 | 1.798 | 0.031 |
| **Location** | |  |  |  | 1.100 | 0.498 |
| Upper outer quadrant | | 70(46.7) | 33(47.1) | 37(52.9) |  |  |
| Upper inner quadrant | | 43(28.7) | 14(32.6) | 29(67.4) |  |  |
| Lower outer quadrant | | 26(17.3) | 10(38.5) | 16(61.5) |  |  |
| Lower inner quadrant | | 5(3.3) | 3(60.0) | 2(40.0) |  |  |
| Subareolar | | 6(4.0) | 3(50.0) | 3(50.0) |  |  |
| **Distance from skin**  (mean ± SD; mm) | | 12.02 ± 4.31  4-28 | 11.29 ± 4.30 | 12.55 ± 4.26 | 0.973 | 0.078 |
| **Number** | |  |  |  | 0.815 | 0.547 |
| Single | | 91(60.7) | 40(44.0) | 51(56.0) |  |  |
| Multiple | | 59(39.3) | 23(39.0) | 36(61.0) |  |  |
| **Shape** | |  |  |  | 1.979 | 0.185 |
| Regular (oval or round) | | 21(14.9) | 6(28.6) | 15(71.4) |  |  |
| Irregular | | 129(85.1) | 57(44.2) | 72(55.8) |  |  |
| **Margin** | |  |  |  | 2.036 | 0.058 |
| Circumscribed | | 46(30.7) | 14(30.4) | 32(69.6) |  |  |
| Uncircumscribed (indistinct/angular/  microlobulated/spiculated) | | 104(69.3) | 49(47.1) | 55(52.9) |  |  |
| **Background echotexture** | |  |  |  | 2.388 | 0.067 |
| Homogeneous | | 27(18.0) | 7(25.9) | 20(74.1) |  |  |
| Heterogeneous | | 123(82.0) | 56(45.5) | 67(54.5) |  |  |
| **Posterior features** | |  |  |  | 0.744 | 0.281 |
| None | | 94(62.6) | 39(41.5) | 55(58.5) |  |  |
| Enhancement | | 28(18.7) | 10(35.7) | 18(64.3) |  |  |
| Shadowing | | 28(18.7) | 14(50.0) | 14(50.0) |  |  |
| **Aspect ratio** | |  |  |  | 2.462 | 0.009 |
| < 1 | | 69(46.0) | 21(30.4) | 48(69.6) |  |  |
| ≥ 1 | | 81(54.0) | 42(51.9) | 39(48.1) |  |  |
| **Calcification** | |  |  |  | 1.612 | 0.019 |
| None | | 86(57.3) | 29(33.7) | 57(66.3) |  |  |
| Coarse calcification | | 30(20.0) | 15(50.0) | 15(50.0) |  |  |
| Microcalcification | | 34(22.7) | 19(55.9) | 15(44.1) |  |  |
| **Adler grade of blood flow** | |  |  |  | 2.422 | 0.010 |
| 0–1 | | 92(61.3) | 31(33.7) | 61(66.3) |  |  |
| 2–3 | | 58(38.7) | 32(55.2) | 26(44.8) |  |  |
| **BI-RADS** **category** | |  |  |  | 1.351 | 0.089 |
| 3 | | 2(1.3) | 1(50.0) | 150.0) |  |  |
| 4a | | 19(12.7) | 4(21.1) | 15(78.9) |  |  |
| 4b | | 42(28.0) | 15(35.7) | 27(64.3) |  |  |
| 4c | | 66(44.0) | 33(50.0) | 33(50.0) |  |  |
| 5 | | 18(12.0) | 10(55.6) | 8(44.4) |  |  |
| 6 | | 3(2.0) | 0(0) | 3(100) |  |  |
| **elastography** | |  |  |  |  |  |
| **Elasticity score** (mean ± SD) | | 4.03 ± 1.01 | 4.43 ± 0.86 | 3.75 ± 1.01 | 2.206 | <0.001 |
| **Elasticity score** | |  |  |  |  |  |
| 1 | | 0(0) | 0(0) | 0(0) |  |  |
| 2 | | 17(11.3) | 4(23.5) | 13(76.5) |  |  |
| 3 | | 22(14.7) | 3(13.6) | 19(86.4) |  |  |
| 4 | | 50(33.3) | 18(36.0) | 32(64.0) |  |  |
| 5 | | 61(40.7) | 38(62.3) | 23(37.7) |  |  |
| **Elasticity score group** | |  |  |  |  |  |
| Low ( < 4) | | 39(26.0) | 7(17.9) | 32(82.1) |  |  |
| High ( ≥ 4) | | 111(74.0) | 56(50.5) | 55(49.5) |  |  |
| **VTI grade** | |  |  |  | 2.173 | <0.001 |
| 1 | | 23(15.3) | 4(17.4) | 19(82.6) |  |  |
| 2 | | 29(19.3) | 7(24.1) | 22(75.9) |  |  |
| 3 | | 32(21.3) | 9(28.1) | 23(71.9) |  |  |
| 4 | 4a | 42(28.0) | 23(54.8) | 19(45.2) |  |  |
|  | 4b | 24(16.0) | 20(83.3) | 4(16.7) |  |  |
| **SWV values**  (mean ± SD; m/s) | | 5.51 ± 2.06 | 6.77 ± 1.57 | 4.59 ± 1.88 | 1.984 | <0.001 |
| **Clinicopathological variable** | |  |  |  |  |  |
| **Age**  (mean ± SD; years) | | 56.94 ± 12.27  (26-92) | 57.05 ± 11.48 | 56.86 ± 12.88 | 1.001 | 0.927 |
| **Age group** | |  |  |  |  |  |
| < 50 | | 43(28.7) | 16(37.2) | 27(62.8) |  |  |
| ≥ 50 | | 107(71.3) | 47(43.9) | 60(56.1) |  |  |
| **Pathological invasive**  **tumor size**  (mean ± SD; mm) | | 25.84 ± 14.47 | 28.43± 16.20 | 23.97 ± 12.86 | 1.849 | 0.013 |
| **Histological grade** | |  |  |  | 3.957 | <0.001 |
| 1 or 2 | | 100(66.7) | 31(31.0) | 6969.0) |  |  |
| 3 | | 50(33.3) | 32(64.0) | 18(36.0) |  |  |
| **Estrogen receptor (ER)** | |  |  |  | 0.975 | 0.941 |
| Negative | | 59(39.3) | 25(42.4) | 34(57.6) |  |  |
| Positive | | 91(60.7) | 38(41.8) | 53(58.2) |  |  |
| **Progesterone receptor (PR)** | |  |  |  | 0.643 | 0.187 |
| Negative | | 81(54.0) | 38(46.9) | 43(53.1) |  |  |
| Positive | | 69(46.0) | 25(36.2) | 44(63.8) |  |  |
| **HER-2** | |  |  |  | 1.186 | 0.636 |
| Negative | | 46(30.7) | 18(39.1) | 28(60.9) |  |  |
| Positive | | 104(69.3) | 45(43.3) | 59(56.7) |  |  |
| **Ki-67 (%)** | |  |  |  | 1.686 | 0.220 |
| ≤ 14 | | 31(20.7) | 10(32.3) | 21(67.7) |  |  |
| > 14 | | 119(79.3) | 53(44.5) | 66(55.5 ) |  |  |
